# A Contemporary Review of Molecular Therapeutic Targets for Adenoid Cystic Carcinoma

**DOI:** 10.3390/cancers14040992

**Published:** 2022-02-16

**Authors:** Lauren E. Miller, Vivienne Au, Tara E. Mokhtari, Deborah Goss, Daniel L. Faden, Mark A. Varvares

**Affiliations:** Department of Otolaryngology—Head and Neck Surgery, Massachusetts Eye and Ear, Boston, MA 02114, USA; viau@meei.harvard.edu (V.A.); tara_mokhtari@meei.harvard.edu (T.E.M.); deborah_goss@meei.harvard.edu (D.G.); dfaden@partners.org (D.L.F.); mark_varvares@meei.harvard.edu (M.A.V.)

**Keywords:** adenoid cystic carcinoma (ACC), salivary gland, targeted therapy

## Abstract

**Simple Summary:**

Adenoid cystic carcinoma (ACC) is a salivary malignancy known for slow growth, a propensity for perineural spread, local recurrence following resection, and indolent distant metastases. Current treatments in recurrent/metastatic (R/M) ACC are generally of limited impact and often palliative in nature. Herein, we review the preclinical and clinical literature on molecular alterations in ACC with the potential for targeted therapeutics. We further review other molecular targets of ongoing investigation and active clinical trials for patients with ACC, offering a contemporary summary and insight into future therapeutic strategies.

**Abstract:**

ACC is a rare malignant tumor of the salivary glands. In this contemporary review, we explore advances in identification of targetable alterations and clinical trials testing these druggable targets. A search of relevant articles and abstracts from national meetings and three databases, including PubMed, Medline, and Web of Science, was performed. Following keyword search analysis and double peer review of abstracts to ensure appropriate fit, a total of 55 manuscripts were included in this review detailing advances in molecular targets for ACC. The most researched pathway associated with ACC is the MYB–NFIB translocation, found to lead to dysregulation of critical cellular pathways and thought to be a fundamental driver in a subset of ACC disease pathogenesis. Other notable molecular targets that have been studied include the cKIT receptor, the EGFR pathway, and NOTCH1, all with limited efficacy in clinical trials. The ongoing investigation of molecular abnormalities underpinning ACC that may be responsible for carcinogenesis is critical to identifying and developing novel targeted therapies.

## 1. Introduction

Adenoid cystic carcinoma (ACC) is a unique malignancy of major and minor salivary glands. Seen in approximately one quarter of all salivary gland malignancies, it is characterized by slow growth, perineural and lymphovascular invasion, and indolent metastasis, most commonly to the lungs [1]. Initial treatment comprises surgical resection, often followed by postoperative radiation therapy; however, local and distant recurrences are common [2,3]. Distant metastasis occurs in an estimated 36–62% of cases [3,4,5,6]. Moreover, many cases of distant metastasis develop without evidence of locoregional disease, making ACC one of the most vexing head and neck malignancies to manage.

Favorable overall survival (OS) rates for patients with ACC have been reported; however, development of distant metastasis significantly lowers both 5- and 10-year OS down to 70% and 29%, respectively [7]. Treatment options for patients with locally advanced, recurrent, or distantly metastatic disease are generally limited and often palliative in nature. In an effort to improve survival and treatment cure rates, single- and combination chemotherapy agents and other systemic agents have been trialed for patients with recurrent/metastatic (R/M) ACC but with limited efficacy [4,8]. In this context, there is an emerging landscape focused on genetic analysis of ACC and improved understanding of the molecular drivers of this disease with the intent to identify potentially targetable mutations for precision treatment options. In this review, we sought to review the landscape of identified and potentially targetable genetic alterations associated with ACC.

## 2. Materials and Methods

We critically reviewed the literature on targeted therapy for salivary adenoid cystic carcinoma using controlled vocabulary and keyword terms. A search of the Ovid MEDLINE (1946–), Embase.com (1947–), and Web of Science Core Collection (1900–) databases was conducted. No limits were applied to restrict date or time period, language or study design. The Preferred Reporting Items for Systematic Reviews and Meta-Analyses for Searching 2021 (PRISMA-S) checklist was used to document the search [9]. All of the searches were designed and conducted by a reference librarian (DG). The final searches were run on 6–7 October 2021. Endnote X7.8 was used to deduplicate the results and Covidence software was used to screen the abstracts and full text documents. Two authors screened the abstracts for inclusion and any discrepancies between screening were sent to a third independent author for final screen. All full text documents were then additionally screened by one author to ensure appropriateness to the study. Please refer to Supplementary File S1 for full details of the search strategy.

## 3. Results

**Figure 1:**
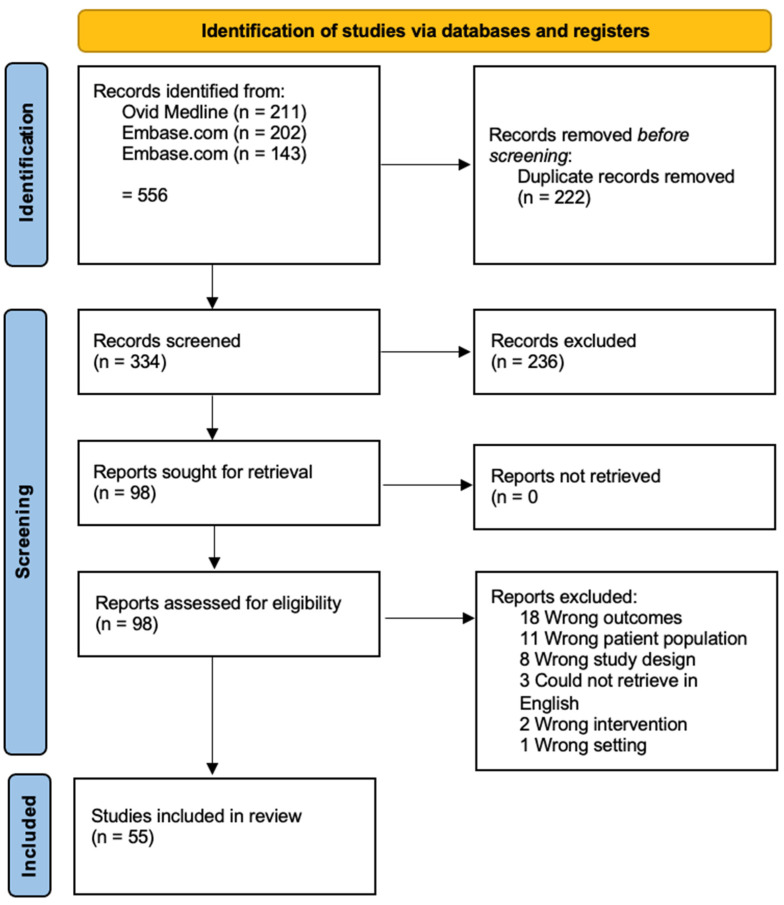
PRISMA flow diagram for ACC database search.

We evaluated 55 manuscripts focused on genomic alterations and therapeutic targets for patients with ACC (Figure 1).

### 3.1. Mutation Burden and Profile

**Figure 2:**
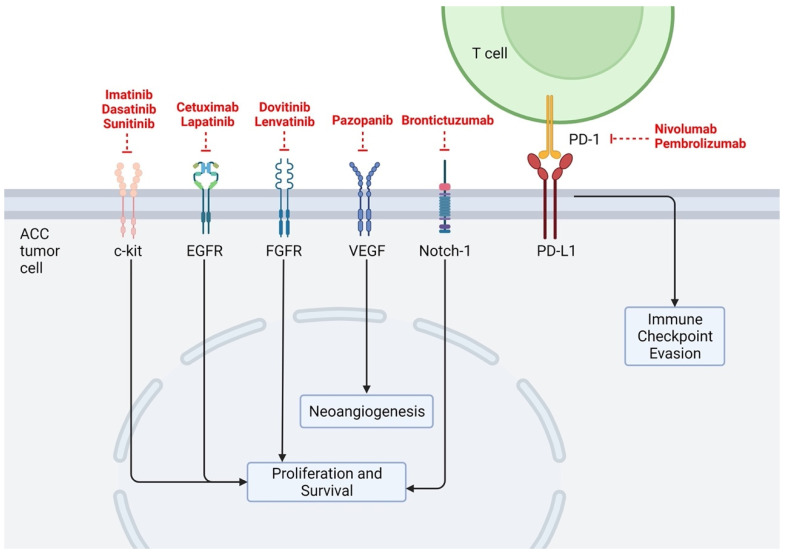
Researched therapeutic targets related to ACC. Created with BioRender.

ACCs have been shown to possess a low mutational burden across numerous studies, matching their indolent clinical behaviors. Using a cohort of 60 tumor samples and performing exome and whole-genome sequencing, Ho et al. found ACCs to have low mutational burden compared to other common head and neck cancers, such as head and neck squamous cell carcinoma (mean 22 coding mutations vs. 130, respectively) [10,11]. This study also identified common alterations in chromatin regulation genes [10], as well as orthogonal pathways such as histone acetyltransferase/deacetylase function, and DNA damage response [11]. Similarly, Rettig et al. studied 25 ACC samples using whole-genome sequencing (WGS) and found a median of 14 mutations per tumor (range 2–36) and again, recurrent alterations in chromatin remodeling genes such as SMARCA2, MLL2, and KDM6A [12]. Potential mutational differences in 1045 ACC tissue samples in patients with primary (*n* = 177) versus recurrent or metastatic (R/M) (*n* = 868) ACC were evaluated using WGS and next-generation sequencing. Notable differences between the two patient groups included significantly increased mutational burden across the NOTCH gene family, chromatin-remodeling genes, tumor suppressor genes, and DNA damage repair genes in R/M cases [13]. Below, we discuss the most common specific mutational targets and possible targeting therapies identified in our analysis (Table 1) as well as their location of action (Figure 2).

### 3.2. MYB

MYB is a transcription factor studied for its known roles in oncogenesis, broadly including cell proliferation and survival [30]. Prior literature has focused on the MYB pathway’s involvement in the ACC mutational landscape, and there is significant evidence for its role in ACC tumorigenesis [31]. Specifically, MYB has been found as a fusion oncogene with nuclear factor 1 transcription family (NFIB) in ACC with a t(6;9)(q23.3;p22.3) translocation [11,13]. In studies characterizing structural variants of ACC using whole-genome sequencing, MYB translocations were the only recurrent structural variants identified, reaffirming its significance [10]. Drier et al. investigated ACC translocations in detail using whole-genome sequencing and demonstrated that repositioning of regulatory elements adjacent to MYB triggered overexpression. This group identified several distinct chromosomal rearrangements, placing super-enhancers adjacent to the MYB locus, and demonstrated that these enhancers directly interact with MYB. Interestingly, when studying how MYB may impact ACC histological classifications (i.e., tubular vs. cribriform vs. solid), unique regulatory and signaling pathways involving TP63 and NOTCH were identified [32].

Another study by Hanna et al. evaluating R/M ACC identified MYB overexpression or rearrangement in 24 of 55 samples [33]. The 10-year overall survival in the MYB-altered subgroup was 100%, the highest of any identified alteration in their cohort [33]. Rettig et al. identified the MYB–NFIB fusion oncogene in 11 of 25 tissue samples. NFIB translocations occurred in 15 of 25 samples, sometimes involving genes besides MYB, suggesting the possible role of NFIB in oncogenesis independent of MYB [12]. However, these samples with NFIB fusions independent of MYB were not confirmed on mRNA expression analysis [12]. In a separate WGS study of eight ACC samples, Thyparambil et al. reported a single sample with fusions of MYBL–NFIB or AHI1–NFIB, in agreement with the aforementioned hypothesis [34,35].

The MYBL1–NFIB fusion is seen less frequently than MYB–NFIB but is similarly known to encourage oncogenic overactivity [36] and thought to have similar oncogenic properties as the MYB–NFIB fusion [37]. In a separate sample with Saida et al., 45 of 52 ACC samples were found to contain translocations in MYB, MYBL1, and NFIB detected via fluorescence in situ hybridization (FISH) [38]. A prior study that found translocations in ACC tumors fusing the MYBL1 gene to the NFIB and RAD51B genes demonstrated similar outcomes to MYB translocations, suggesting a potential interchangeable nature to these drivers of ACC [39].

Despite the high rate of translocation and mutation of MYB in ACC, actionable targets acting along this pathway have had little success. There has been some discovery in vitro research to understand potential targets to the MYB pathway. Recent work by Yusenko et al. evaluated a MYB inhibitory compound, Bcr–TMP, that acts as a highly active MYB inhibitory compound, demonstrating anti-proliferative effects on ACC cells [40]. This same group further identified inhibition of MYB through proteasome inhibitors; further analysis of one such proteasome inhibitor, oprozomib, interfered with MYB stimulatory activity [41]. Hanna et al. investigated the use of Tretinoin (all-trans retinoic acid) in patients with R/M ACC, as the retinoic acid receptor has been suggested to play a role in the downregulation of MYB expression in studies on myeloid leukemia. The study reported no response in 18 patients and a median progression-free survival of 3.2 months [29]. Andersson et al. identified that IGF1R/AKT inhibition downregulated MYB–NFIB activity in ACC models, suggesting a potential strategy to target transcriptional regulation in ACC [42]. His group more recently identified the DNA-damage sensor kinase ATR as a downstream therapeutic target of MYB that appears to be overexpressed in primary ACCs. Further, treatment with an ATR kinase inhibitor (VX-970) demonstrated a dose-dependent decrease in proliferation and induced apoptosis in MYB-positive ACC cells. This study demonstrates the first downstream MYB effector that has been acted upon as a therapeutic target, which, given the ubiquity of MYB dysregulation in ACC, opens the door for further investigation and potential therapeutic intervention [37].

### 3.3. cKIT

cKIT is a receptor tyrosine kinase involved in intracellular signaling and cellular deregulation with known roles in the development of leukemia, melanoma, thyroid cancer, and breast cancer [43]. The role of cKIT in ACC has been extensively studied. Many patients with ACC have demonstrated overexpression of cKIT, thought to range from 60 to 90% of ACC tumors [44,45]. A study by Vila et al. in 2009 was the first to examine the cKIT gene mutation in primary ACC, with cKIT missense point mutations detected in seven of eight samples (88%) [46]. The identification of gain-of-function mutations in exon 11, and less frequently in exons 9, 13 and 17, suggested tyrosine kinase inhibitors as a potential treatment for ACC. Copy number variations in cKIT have also been investigated. Freier et al. performed fluorescent in situ hybridization (FISH) on ACC samples found to express cKIT. In this cohort, 6% of ACC tumors demonstrated a cKIT copy number gain, suggesting that a gain in gene copy number may explain increased cKIT protein expression in a limited subset of ACC pathogenesis [47].

Targeted therapies against the cKIT receptor using tyrosine kinase inhibitors were one of the first attempts of precision therapy in patients with ACC, specifically using imatinib, dasatinib, or sunitinib therapy. Unfortunately, while high cKIT expression in ACC has been well established, these studies were largely disappointing, with an overall response rate (ORR) below 5% and no significant improvement in patient survival in any of these trials [14,15,17,18].

In a phase II trial for imatinib, ten patients with advanced or metastatic ACC cKIT-positive tumors were enrolled in daily dosing of imatinib at 400 mg/day, without any responses seen in the study, and eight of the ten with disease progression after a median of 6 months [14]. In a separate phase II trial, no objective responses were appreciated in 15 patients with the same dosing of imatinib [15]. A separate study explored the efficacy of imatinib with cisplatin for patients with ACC with a known overexpression of cKIT. Response to treatment was followed with imaging, demonstrating a partial response in 3 of 28 patients and 19 of 28 patients with stable disease [16]. Notably, their cohort overall survival was 35 months.

Wong et al. studied the efficacy of dasatinib in 40 patients with ACC and cKIT-positive tumors determined by immunohistochemistry [17]. Only one objective response (2.5%) was reported. Twenty patients (50%) had stable disease and 29 eventually experienced disease progression. Mean progression-free survival was 4.8 months. Median survival for this cohort was 14.5 months, with a six-month survival rate of 81.5%.

In the trial studying response of R/M ACC patients to daily sunitinib, there were no objective responses in 13 patients, with median OS of 18.7 months [18]. These largely disappointing findings have led many investigators to conclude that the cKIT pathway is not a primary driver in ACC tumorigenesis.

### 3.4. EGFR

Epidermal growth factor receptor (EGFR) is a transmembrane tyrosine kinase receptor which, when activated, stimulates mitosis and leads to cell proliferation. EGFR is overexpressed in a variety of tumors and it is thought to be overexpressed in up to 85% of ACC, making it a therapeutic target of interest [19]. The immunohistochemical expression of EGFR in ACC tumors has been characterized; in a study of 25 ACC samples, EGFR expression was quantified as weak-moderate in 32% and as strong in 64% of samples [48].

Prior studies have targeted the EGFR pathway via numerous therapeutic agents including gefitinib [49], cetuximab [19], and lapatinib [20] without meaningful response rates in previously treated patients. Specifically, for cetuximab, no patients with ACC (*n* = 23) demonstrated a meaningful response, with 12 of 23 patients having disease stabilization greater than 6 months [19]. In another study of 19 patients with advanced ACC, lapatinib [20], a small molecule with dual EGFR and erbB2 tyrosine kinase activity, was studied. No responses were observed in this patient population; 15 patients demonstrated disease stabilization of 6 months or greater.

### 3.5. FGFR

Fibroblast growth factor receptor 1 (FGFR1) is a downstream pathway from the MYB gene and upregulation can lead to overexpression of FGF in patients with ACC [50]. Dovitinib, a small molecular inhibitor of FGFR1, was assessed for possible therapeutic effect in patients with advanced ACC and had a partial response rate and disease stabilization rate of 6% and 65%, respectively [21,51]. A majority (67%) of patients with disease stabilization eventually developed progressive disease in this study; the overall median progression-free survival was 8.5 months.

Lenvatinib is a new-generation multi-kinase inhibitor against FGFR1-3, VEGFR2, cKIT, RET and PDGFR alpha and beta, and has been found to have more promising results [51]. To date, two separate studies by Locati et al. and Tchekmedyian et al. have investigated the efficacy of lenvatinib in R/M ACC. Locati et al. evaluated 26 patients in their cohort, noting a partial response rate of 12% (*n* = 3). In patients with stable disease (*n* = 20), tumor shrinkage by radiographic evaluation was reported to be approximately 25% in 4 patients. Dose adjustment was required in the vast majority of patients (92%). The median progression-free survival and OS were 9.1 months and 27 months, respectively [22]. Similarly, Tchekmedyian et al. reported a partial response rate of 15.6% in 32 patients with R/M ACC. Eight (25%) patients had more than 20% reduction in tumor size [23]. At least one dosage modification was required in 72% of patients. The median progression-free survival was reported to be 17.5 months.

In light of these results, lenvatinib was designated a National Comprehensive Cancer Network (NCCN) grade 2b [52] recommendation (NCCN panel vote of at least 50–85%), for treatment of progressive or R/M ACC in the NCCN Head and Neck Cancers guidelines V.1.2020 [53]. In an ongoing phase II study, the efficacy of combination lenvatinib and pembrolizumab therapy in treating advanced ACC and other salivary gland cancers is currently underway, with the study completion date estimated to be December 2022 [54].

### 3.6. VEGF

Vascular endothelial growth factor (VEGF) has been assessed for a potential role in ACC disease progression, given the role of other growth factors as described above as well as its role in tumor angiogenesis. In prior studies assessing ACC tumor samples, VEGF expression was considered a poor prognostic factor for tumor stage [55] as well as overall survival [56,57].

Pazopanib, a small molecule inhibitor of VEGFR, PDGFR, and KIT, has previously been assessed for antitumor activity in ACC patients with underwhelming results, including 1 of 46 patients demonstrating a partial response and 35 of 46 with stable disease [24]. The median progression-free survival and overall survival were reported at 5.9 months and 16.6 months, respectively.

### 3.7. NOTCH1

The NOTCH signaling pathway is a well-known critical regulator of cell proliferation and survival. In particular, mutation of the NOTCH1 gene has been shown to play a role in R/M ACC, possibly via induction of neoangiogenesis as the mechanism of tumor growth [58]. Further, NOTCH1 has been found to be heavily mutated across ACC [13]. Patients who exhibited NOTCH1 mutations in ACC samples have been associated with an overall shorter survival [59]. Activating NOTCH1 is present in approximately 20% of ACC and is associated with a more aggressive disease course with higher rates of bone and liver metastases [60]. Chintakuntlawar et al. reviewed genetic testing results for 23 ACC patients and identified 41 unique mutations, among which 22% (5/23) demonstrated NOTCH mutations [61].

Additional literature has suggested increased rates of NOTCH mutation in R/M cases. For example, Ho et al. demonstrated a significantly higher proportion of NOTCH1 mutations in R/M cases of ACC compared to primary cases [13]. Similarly, Su et al. noted higher expression levels of NOTCH1 in R/M ACC cases as compared to primary ACC tumors [62]. Activated NOTCH1 upregulates genes such as BCL-2 and CCND1, well-known anti-apoptotic and cell cycle-related genes, which suggests a possible role of NOTCH1 in metastatic ACC.

Mouse models with NOTCH1 mutants receiving specific monoclonal antibodies targeting NOTCH1 have demonstrated partial responses in ACC tumor size [60]. However, human phase I trials have demonstrated minimal responses, with ranging therapeutic toxicities [63]. A phase I trial studying bronticuzumab, the monoclonal antibody against Notch1, demonstrated 6 of 36 ACC subjects had either a partial or prolonged period of disease stabilization (NCT01778439) [25,64]. A phase I trial of the small-molecule CB-103, an upstream inhibitor of the NOTCH pathway, is also being studied with preliminary data demonstrating a median progression-free survival of 22 weeks in ACC patients (NCT03422679) [65]. The preliminary results from the ACCURACY trial, an open-label, multicenter study of AL101, a small molecule selective gamma-secretase inhibitor that blocks Notch signaling, demonstrated early disease activity with a response rate of 15% [66]. The study is estimated to complete in December 2022.

### 3.8. Estrogen Receptor

Several studies have investigated the possibility of estrogen receptor blockade as a therapeutic target for ACC. Estrogen receptor (ER)-beta subtype has been found to be expressed in salivary gland cells, and it is hypothesized that estrogen may regulate salivary gland physiology [67]. Another report found significantly increased ER-beta nuclear expression in 32 of 38 cases of ACC [68]. However, to date, it is unclear what role increased ER-beta subtypes plays with regard to ACC tumorigenesis, or the feasibility of estrogen receptor blockage as a therapeutic target for ACC.

### 3.9. PI3K/PTEN/mTOR Pathway

The PI3K/PTEN/mTOR pathway is a complex pathway that transmits proliferative intracellular signals from membrane-bound receptors [69]. Various components of this pathway have been explored for their roles in ACC pathogenesis. In a phase II study of everolimus, an mTOR receptor inhibitor, to treat progressive unresectable ACC, everolimus showed promising efficacy. Among 34 enrolled patients, median progression-free survival was 11.2 months with 27 patients showing stable disease and tumor shrinkage seen in 15 subjects [70]. Results from a recent phase I study investigating the combined effects of lenalidomide in combination with everolimus demonstrated this combination to be safe and tolerable with particular excitement for their efficacy in ACC [71].

The FGF/IGF/PI3K pathway has been a target for prior study in ACC, with Ho et al. identifying recurrent mutations in 30% of ACC tumors in this pathway [10].

PTEN has been studied extensively as one of the most important tumor suppressors in human cancers; inhibition of PTEN promotes tumorigenesis. Liu et al. reported that loss of PTEN expression was most frequently seen in ACC as compared to other salivary gland malignancies, representing 26 of 55 of ACC samples, especially in the poorly differentiated, high-grade subtype of solid ACC (18/22) [72].

Yu et al. found the expression of p-S6 (downstream molecule of mTOR), p-Stat3, PAI, EGFR and HIF-1a was significantly increased in 72 ACC samples, compared with 12 pleomorphic adenoma and 18 normal salivary glands tissues, suggesting possible mTOR downstream inhibitors as ACC therapy targets [73].

In mouse models with overexpression of the serine/threonine kinase AKT3, a downstream target of the PI3K pathway, Zboray et al. found that AKT3 overexpression led to ACC with 100% penetrance, while reversal of its expression could revert the phenotype, suggesting a potential therapeutic target in preclinical models [74].

### 3.10. CDK/Cell Cycle

Cyclins and cyclin-dependent protein kinases promote cell proliferation. Cyclin-dependent protein kinase 6 (CDK6) inactivates the retinoblastoma protein (Rb), a major protein for arresting the cell cycle at G1 [75]. CDK6 expression was four times higher by mass spectrometry in ACC samples (*n* = 8) than SCC samples (*n* = 6) and three-fold higher at the mRNA level [35]. In addition, the expression of p16 protein, an inhibitor of CDK6, was three-fold lower in ACC than in SCC and all ACC samples harbored intact retinoblastoma (RB1) gene. The combination of these results suggests that ACC may respond to treatment with CDK6 inhibitors [34].

### 3.11. Tumor Microenvironment and Immune Checkpoint Targets

Checkpoint inhibitors and cancer immunotherapy have revolutionized cancer treatment in recent years, focusing on the development of therapies to enhance endogenous antitumor immune response [36]. Checkpoint inhibitor therapy has had limited success for patients with ACC. The NISCAHN trial evaluated the programmed cell-death receptor 1 (PD-1) monoclonal antibody nivolumab in 45 R/M ACC patients [26]. In this cohort, 33% had progression-free survival at 6 months with an overall response rate of 8.8%. Another trial of nivolumab with CTLA4 monoclonal antibody ipilimumab showed an overall response rate of 6% (2 of 32 patients), despite substantial responses in tumor reduction on imaging (73.1% and 58.4%) [76]. In a study of 20 patients with ACC comparing pembrolizumab with or without hypofractionated radiotherapy, there was no evidence of tumor response [77].

There has been significant effort investigating the role of PD-L2 and the tumor microenvironment in ACC. Sridharan et al. found PD-L2 expression in 9 of 15 primary and 8 of 11 metastatic ACC tumor deposits [78]. Forty-two percent of both samples contained “many” immune cells, defined as >100 per high power field. They also found higher PD-L2 expression was associated with lower immune infiltrates across all cell types, including NK cells, CD4+, and CD8+ T cells. Notably, PD-1 expression was absent in all ACC deposits. The authors posit this finding may lead to a potential role for PD-L2 inhibition as treatment for patients with ACC.

Mosconi et al. corroborated these findings in their investigation of levels of PD-L1, PD-L2, PD-1, and CTLA-4, as well as markers of tumor-infiltrating lymphocytes and dendritic cells in 36 ACC samples [79]. The greatest immunohistochemical expression in ACC samples was PD-L2 and HLA-G, an immune inhibitory molecule. Similarly, no samples expressed PD-L1. Overall, these studies suggest low immunogenicity in the ACC microenvironment of the samples studied.

### 3.12. Other

Protein arginine methyltransferase 5 (PRMT5) has been investigated in relation to ACC. In vitro investigation has shown that PRMT5 inhibition decreases expression of MYB and associated downstream genes; it has been seen to significantly inhibit the growth of ACC in vivo [80]. The PRMT5 inhibitor GSK3326595 has shown a response rate of 21% (3/14) in an early trial that included 14 subjects with ACC [28].

A Dutch study is currently evaluating Lutetium-177 (a radiolabeled prostate-specific membrane antigen (PSMA)-binding small molecule) given initial findings that 90% of ACC expresses PSMA with positive uptake on PSMA-focused PET scans. The study is currently in process with a completion goal in 2025 (NCT04291300) [81].

A full list of previously researched therapeutic targets is depicted in Figure 1. Ongoing active clinical trials are detailed in Table 2.

#### Preclinical Studies

Unfortunately, preclinical target discovery has been challenging in large part due to the difficulty to grow and maintain ACC cell lines [37]. This is due to a combination of intratumor heterogeneity in ACC disease, unclear biologic tumor etiology, and long and currently unvalidated cell line development. Prior work has shown successful growth of recapitulated tumor in xenograft models derived from the donated ACC tumor 74% of the time [82]. Wang et al. similarly used tumor samples from human salivary ACC in immunodeficient mice to establish patient-derived xenografts; this model primarily demonstrated (53.9%) the MYB/MYBL1–NFIB fusion mutation. They evaluated both a Pi3K inhibitor and retinoic acid, with inhibition in those tumors harboring either PIK3CA mutation or MYB–NFIB, respectively [83].

However, these patient xenograft models can be prohibitively expensive, with a low success rate and an extended time required to obtain results [84]. Another group established ACC cell cultures from patient-derived xenograft tumors utilizing conditional reprogramming, with an in vivo zebrafish model to evaluate the MYB translocation. They subsequently tested regorafenib, a multi-kinase small molecule inhibitor that targets both VEGFR2 and tyrosine kinase receptors. There was overall greater tumor growth inhibition in the treatment model compared to controls [84]. Although more research is needed to fully understand whether in vivo models reflect primary tumor genotypes and phenotypes, these preclinical models generate promise for further research.

## 4. Discussion

ACC is a common salivary neoplasm of the head and neck region and is notoriously characterized by its predilection for delayed recurrence and development of distant metastasis after long periods of quiescence [85]. Long-term survival following initial disease management has been previously reported as 77% at five years [86], with survival rates of distantly metastatic ACC dropping to 32% at five years [87]. As such, the treatment of ACC is particularly challenging, even in cases of excellent initial treatment response, and ongoing long-term surveillance is a critical component of oncologic care for this patient population. Further, traditional chemotherapeutic agents have demonstrated poor efficacy in managing advanced, recurrent, or metastatic ACC. In this context, there has been significant interest in recent years to study targeted therapies that may offer improved outcomes.

Herein, we provide a comprehensive review of the explored molecular targets for adenoid cystic carcinoma. The most well-studied pathway associated with ACC is the MYB–NFIB translocation, considered by many to represent a signature molecular event in ACC oncogenesis. This translocation gives rise to dysregulation of the MYB gene and subsequently constitutive activation of downstream genes, leading to dysregulation of critical cellular pathways involved in apoptosis, cell adhesion, and cell cycle regulation [88]. Interestingly, MYB overexpression has been identified in ACC samples that are negative for the MYB–NFIB fusion suggesting additional genetic mechanisms may also drive this disease [89]. In vitro immunohistochemical analysis of both fusion-positive and fusion-negative human ACC tissue samples performed using a monoclonal antibody against the N-terminal domain of human MYB demonstrated strong nuclear staining for MYB in fusion-positive tumors (85%) but also in fusion-negative tumors (61%) [89]. Given the frequency of the MYB–NFIB translocation and its presumed role as a fundamental driver in a subset of ACC disease pathogenesis, significant ongoing focus on this molecular finding may provide a fertile target for potential therapeutics.

Therapies directed against the cKIT receptor have historically been some of the earliest therapeutic targets for directed therapy against ACC. While cKIT expression is known to be high in ACC tumors, multiple independent studies evaluating tyrosine kinase inhibitors of cKIT such as imatinib have been largely disappointing [46]. Therapeutics targeting the EGFR pathway (gefitinib, cetuximab, and lapatinib) have been some of the most investigated but similarly have demonstrated little efficacy against ACC [19,20,49]. Two recent studies of lenvatinib, a kinase inhibitor acting on the VEGFR, PDGFR, FGFR, KIT, and RET pathways, have shown exciting results in reducing tumor burden in patients with recurrent/metastatic ACC [22,23]. There are several ongoing clinical trials investigating novel applications of immunotherapy in ACC. To date, nearly all therapeutic trials have shown lackluster results. However, significant research into targeted immunotherapies and the understanding of ACC pathogenesis are rapidly evolving.

Next-generation sequencing techniques are helping to identify molecular abnormalities that underpin ACC pathogenesis, the first step to the development of specific targeted therapies. Recent whole exome and genomic sequencing of ACC tumors has pushed our understanding of this disease. An ever-expanding number of ACC tumors are being sequenced, with data suggesting genomic alterations in several pathways (i.e., MYB transcriptional activator family, EGFR/KRAS pathway, chromatin remodeling, tyrosine kinase signaling, and DNA damage/checkpoint signaling) as potential areas that may be amenable to directed therapeutic action [12,13,48,90].

Recent advances in our understanding of disease-specific chromosomal and genetic fingerprints in ACC offer exciting potentials for therapeutic interventions and prognosis. The ongoing investigation of molecular abnormalities underpinning ACC that may be responsible for carcinogenesis is critical to identifying and developing novel targeted therapies. As pertains to this review, ACC is focused on tumors arising within salivary tissue; however, it is important to recall that ACC tumors may also originate in the trachea, lung, and breast, among others [91]. Therefore, advances in the understanding of the molecular pathogenesis and associated possible targeted therapeutics for salivary ACC may offer insights into ACC elsewhere in the body.

## 5. Conclusions

Traditional therapeutic agents for patients with advanced, recurrent, or metastatic ACC have demonstrated poor efficacy in prolonging survival. There has been significant focus on identification and development of targeted therapies that may potentially improve outcomes. The most researched pathway associated with ACC is the MYB–NFIB translocation, found to lead to dysregulation of critical cellular pathways and thought to be a fundamental driver in a subset of ACC disease pathogenesis. Other notable molecular targets that have been studied include the cKIT receptor, the EGFR pathway, and NOTCH1, all with limited efficacy in clinical trials. Ongoing research on potential targeted therapies for ACC is critical to identifying and developing novel targeted therapies.

## Figures and Tables

**Table 1:** Molecular targets previously studied in ACC.

Targeted Molecule/Pathway	Author; Year	Therapeutic/Drug	Patients *n*	Complete Response *n* (%)	Partial Response *n* (%)	Stable Disease *n* (%)	Progressive Disease *n* (%)	mPFS (mo)	mOS (mo)	Ref.
cKIT	Pfeffer et al.; 2007	Imatinib	10	0 (0)	0 (0)	2 (20)	5 (50)	6 *	-	[14]
	Hotte et al.; 2005	Imatinib	15	0 (0)	1 (7)	2 (13)	6 (40)	2.5	7.5	[15]
	Ghosal et al.; 2011	Imatinib + Cisplatin	28	0 (0)	3 (11)	19 (68)	4 (14)	15	35	[16]
	Wong et al.; 2016	Dasatinib	40	0 (0)	1 (3)	20 (50)	12 (30)	4.8	14.5	[17]
	Chau et al.; 2012	Sunitinib	13	0 (0)	0 (0)	11(85)	2 (15)	7.2	18.7	[18]
EGFR	Locati et al.; 2009	Cetuximab	23	0 (0)	0 (0)	20 (87)	3 (13)	-	-	[19]
	Agulnik et al.; 2007	Lapatinib	19	0 (0)	0 (0)	15 (79)	9 (53)	3.5	NR	[20]
FGFR	Dillon et al.; 2017	Dovitinib	34	0 (0)	2 (6)	22 (65)	1 (3)	8.2	20.6	[21]
	Locati et al.; 2020	Lenvatinib	26	0 (0)	3 (12)	20 (77)	3 (12)	9.1	27	[22]
	Tchekmedyian et al.; 2019	Lenvatinib	32	0 (0)	5 (16)	24 (75)	1(3)	17.5	-	[23]
VEGF	Guigay et al.; 2016	Pazopanib (VEGFR, PDGFR, KIT)	46	0 (0)	1 (2)	35 (76)	10 (22)	5.9	16.6	[24]
NOTCH1	Ferrarotto et al.; 2018	Brontictuzumab	NS	0 (0)	2 (NS)	3 (NS)	-	9.9	-	[25]
PD-1	Fayette et al.; 2019	Nivolumab	45	0 (0)	4 (9)	26 (57)	-	4.9	-	[26]
	Mahmood et al.; 2021	Pembrolizumab +/− radiation therapy	10	0 (0)	0 (0)	5 (50)	2 (50)	4.5	NR	[27]
		Pembrolizumab	9	0 (0)	0 (0)	7 (78)	2 (22)	6.6	27.2	
PRMT5	Siu et al.; 2019	GSK3326595	14	-	3 (21)	-	-	-	-	[28]
ATRA	Hanna et al.; 2021	Tretinoin	16	0 (0)	0 (0)	11 (69)	5 (28)	3.7	-	[29]

Abbreviations: mPFS, median progression-free survival; mOS, median overall survival; NS, not specified; * mean, not median.

**Table 2:** Ongoing active clinical trials related to ACC.

Study Title	Intervention	Molecular Targets	Status
Lenvatinib and Pembrolizumab in people with Advanced Adenoid Cystic Carcinoma and other Salivary Gland Cancers [48]	Lenvatinib Pembrolizumab	FGFR1-3, VEGFR2, cKIT, RET and PDGFR alpha and beta, PD-1	Recruiting
Amivantamab in Adenoid Cystic Carcinoma [76]	Amivantamab	EGFR-ET	Not yet recruiting
9-ING-41 Plus Carboplatin in Salivary Gland Carcinoma [77]	9-ING-41 Carboplatin	GSK-3	Recruiting
Lutetium-177-PSA Radioligand Therapy in Advanced Salivary Gland Cancer Patients [26]	Lutetium-177-PSMA-I&T	PSMA	Recruiting
APG-115 in Salivary Gland Cancer Trial [78]	APG-115 Carboplatin	p53	Recruiting
Nivolumab and Ipilimumab in Treating Patients With Rare Tumors (Including ACC)	Ipilimumab Nivolumab	CTLA-4PD-1	Recruiting

## Supplementary Materials

The following supporting information can be downloaded at: https://www.mdpi.com/article/10.3390/cancers14040992/s1, Supplementary File S1: Database Search Terms.
