# Supplementary material for: A Contemporary Review of Molecular Therapeutic Targets for Adenoid Cystic Carcinoma
Source: Cancers (Basel). 2022 Feb 16;14(4):992. doi: 10.3390/cancers14040992 (PMC8869877; doi:10.3390/cancers14040992)
Supplement: Supplementary file 1 [file cancers-14-00992-s001.zip › cancers-1568644-supplementary.pdf]

Supplementary file S1: Database Search Terms

Embase

10/6/2021

(proteogenom* OR genetic* OR genom* OR 'gene* mutation*' OR exome OR molecule* OR mutat* OR inhibitor*) NEAR/15 (therap* OR treatment*)

589,388

AND

'salivary adenoid cystic carcinoma'/exp OR 'salivary adenoid cystic carcinoma':ti,ab OR 'salivary gland tumors':ti,ab OR 'salivary gland tumours':ti,ab OR 'secretory gland*':ti,ab OR 'salivary gland carcinoma*':ti,ab OR 'salivary gland cancer*':ti,ab

5,882

OR

saliv* NEAR/3 'adenoid cystic carcinoma*'

1,058

2 OR 3 = 6,304

1 & 4 = 202

Medline

10/6/2021

(proteogenom* OR genetic* OR genom* OR 'gene* mutation*' OR exome OR molecule* OR mutat* OR inhibitor*) adj15 (therap* OR treatment*)

752,830

"salivary adenoid cystic carcinoma".ti,ab. OR "salivary gland tumors".ti,ab. OR "salivary gland tumours".ti,ab. OR "secretory gland*".ti,ab. OR "salivary gland carcinoma*".ti,ab. OR "salivary gland cancer*".ti,ab.

4,466

OR

saliv* adj3 "adenoid cystic carcinoma*"

800

2 OR 3 =4860

1 & 4 = 211

Web of Science Core Collection:

Science Citation Index Expanded (SCIE) -1900 to present.

Social Sciences Citation Index (SSCI) - 1900 to present.

Arts & Humanities Citation Index (AHCI) - 1975 to present.

Emerging Sources Citation Index (ESCI) - 2005 to present.

Conference Proceedings Citation Index (CPCI) - 1990 to present.

Book Citation Index (BKCI) - 2005 to present.

Current Chemical Reactions and Index Chemicus

10/7/2021

(proteogenom* OR genetic* OR genom* OR "gene* mutation*" OR exome OR molecule* OR mutat* OR inhibitor*) NEAR/15 (therap* OR treatment*)

391,746

"salivary adenoid cystic carcinoma" OR "salivary gland tumors" OR "salivary gland tumours" OR "secretory gland*" OR "salivary gland carcinoma*" OR "salivary gland cancer*"

5064

OR

saliv* NEAR/3 "adenoid cystic carcinoma*"

843

2 OR 3 = 5505

1 AND 4 = 143
